# Impact of Neoadjuvant Chemotherapy on Clinical Risk Scores and Survival in Patients with Colorectal Liver Metastases

**DOI:** 10.1245/s10434-016-5615-3

**Published:** 2016-10-11

**Authors:** Kerstin Wimmer, Christoph Schwarz, Carmen Szabo, Martin Bodingbauer, Dietmar Tamandl, Martina Mittlböck, Klaus Kaczirek

**Affiliations:** 1 Department of General Surgery, Medical University of Vienna, Vienna, Austria; 2 Department of Biomedical Imaging and Image-Guided Therapy, Medical University of Vienna, Vienna, Austria; 3 Center for Medical Statistics, Informatics and Intelligent Systems, Section for Clinical Biometrics, Medical University of Vienna, Vienna, Austria

## Abstract

**Background:**

Several clinical risk scores for patients with colorectal liver metastases (CLM) were established in cohorts of patients undergoing liver resection (LR) without neoadjuvant chemotherapy (NAC). The purpose of the study was to evaluate the predictive values of four common risk scores in the setting of NAC and the impact of score changes during NAC.

**Methods:**

Risk scores (Fong, Nordlinger, Nagashima, and Konopke) were retrospectively calculated for 336 patients undergoing LR for CLM, including 109 patients without and 227 patients with NAC. In patients with NAC, the scores were calculated before and after NAC.

**Results:**

In patients without NAC (*n* = 109), all risk scores except the Konopke score showed a significant correlation with disease-free survival (DFS). Only the Nagashima score also was predictive for overall survival (OS). In patients with NAC (*n* = 227), all scores except the Konopke score were predictive for DFS and OS before and after NAC. Score changes in the Fong and the Nagashima score showed a significant correlation with DFS and OS.

**Conclusions:**

Nagashima score was the most universally applicable score and predicted prognosis in all tested scenarios.

Survival of patients with colorectal liver metastases (CLM) largely depends on their chance to undergo potentially curative surgical resection.

However, prognosis and outcome are determined by a wide range of patient- and tumor-dependent variables, including tumor size, lymph node positivity of the primary tumor, synchronous metastases, and elevated carcinoembryonic antigen amongst others^1^. To predict prognosis after liver resection of CLM, several clinical risk scores have been developed. The most commonly used scores are the clinical risk score (CRS) by Fong, Nordlinger, Nagashima, and Konopke^2^–^5^. All of these scores were established in cohorts of patients undergoing liver resection without prior chemotherapy.

Neoadjuvant chemotherapy (NAC) was introduced to prolong progression-free survival in upfront resectable metastases and to achieve secondary resectability in borderline or nonresectable metastases^6^–^8^. NAC is now widely used in clinical practice.

The increasing number of patients receiving NAC might impede the general application of risk scores in the clinical practice^9^. Furthermore, it remains unclear whether a change in risk scores during NAC impacts on survival. To clarify these issues, we analyzed a series of 336 patients undergoing liver resection (LR) for CLM consisting of 227 patients who received NAC and of 109 patients who did not.

## Materials and Methods

This is a retrospective analysis of 336 patients who underwent liver resection for CLM at our institution from May 2000 to December 2010. Data were extracted from a prospective patient database, laboratory records, and patients charts. The study was approved by the local ethics committee at the Medical University Vienna, Austria. Relevant data for this study included: demographic data, number and size of metastases, primary tumor and lymph node stage, interval from diagnosis of primary tumor to detection of metastases, carcinoembryonic antigen (CEA) levels, resectability of any extrahepatic disease, type of NAC, as well as overall (OS) and disease-free survival (DFS).

Patients were divided into two groups depending on whether they received NAC or not. Easily resectable, small liver metastases with a good prognosis or patient’s wish formed the basis of the decision of omitting NAC. Standard duration of NAC was 3 months in patients with primarily resectable metastases or until resectability in patients with initially unresectable CLM. Resectable CLM are defined as liver metastases, in which upfront R0 resection of all hepatic lesions is possible, more than 30 % estimated residual liver after resection and disease not in contact with major vessels of the remnant liver. Clinical scores were calculated before and after NAC, and change was classified as decreasing, steady, or increasing scores.

### Prognostic Scores

The following scores were calculated and compared: the CRSs by Fong, Nordlinger, Nagashima, and Konopke^2^–^5^. The detailed description of each CRS is shown in Table 1. Based on the original publication of Fong et al., we used the original stratification of two risk groups. All other scores defined three risk groups^2^.

**Table 1:** Clinical risk scores

	CRS criteria (1point for 1 risk factor)	Risk groups
Fong	1. Largest liver metastasis >5 cm 2. Disease free interval <12 months 3. Number of liver metastases >1 4. Lymph node positive primary tumor 5. CEA >200 ng/ml	*Low* 0–2 pts *High* 3–5 pts
Nordlinger	1. Age >60 a 2. Serosal invasion of primary tumor (≥pT3) 3. Lymph node positive primary tumor (pN1) 4. Disease free interval <24 months 5. Number of liver metastases >3 6. Largest liver metastasis >5 cm	*Low* 0–2 pts *Intermediate* 3–4 pts *High* 5–6 pts
Nagashima	1. Serosal invasion of primary tumor (≥pT3) 2. Lymph node positive primary tumor (pN1) 3. Number of liver metastases ≥2 4. Largest liver metastasis ≥5 cm 5. Resectable extrahepatic metastases	*Low* 0–1 pts *Intermediate* 2–3 pts *High* ≥4 pts
Konopke	1. Number of liver metastases ≥4 2. CEA ≥200 ng/ml 3. Synchronous liver metastases	*Low* 0 pts *Intermediate* 1 pt *High* ≥2 pts

In patients without NAC, scores were calculated before surgery. In patients with NAC, scores were calculated before the start of NAC and immediately before liver resection.

Scores were only calculated when all relevant parameters were available. Patients with missing data were excluded for the respective score calculation. Patients with NAC were only included in statistical analysis of survival curves if the clinical risk scores were available for both time points of evaluation. Furthermore, the differences between the scores before and after the administration of NAC were calculated to show if hypothesized prognostic improvement or decline would cause changes in survival curves. Any reduction of the score number was classified as −1, no change as 0, and any increase as +1.

### Statistical Analysis

Statistical analysis was performed using SPSS for Windows, Version 20.0 (SSPS Inc., Chicago, IL). Metric variables were expressed as mean or median ± standard deviation. The comparison of variables before and after NAC was performed by the paired *t* test if the differences between the values before and after NAC were normally distributed. If not, the Wilcoxon signed-rank test was used. Survival curves were computed with a Kaplan–Meier graph and compared with the log-rank test. In case of risk scores with three risk groups, the log-rank test was combined subsequently with a linear trend test. For comparison of survival curves of patients with a score difference between before and after NAC of −1, 0, or +1, the Kaplan–Meier method was combined with a linear trend test. A *p* value <0.05 was considered to indicate statistical significance.

## Results

A total of 336 patients undergoing liver resection for CLM were included in the study including patients without (*n* = 109) and with NAC (*n* = 227). Patient characteristics are shown in Table 2. The median age of the study population was 63.0 (range, 28–87) years, and the median follow-up was 42.0 (range, 0–140) months. Between patients with and without NAC, no statistically significant difference but a trend towards longer survival in patients without NAC became apparent. Patients without NAC had a longer DFS (14 vs. 9 months, *p* = 0.206) and OS (54 vs. 50 months, *p* = 0.584) than patients receiving NAC.

**Table 2:** Characteristics of all patients

	All patients (*n* = 336)		Patients without NAC (*n* = 109)		Patients with NAC (*n* = 227)	
	Value	%/Range	Value	%/Range	Value	%/Range
Male	214	64	76	70	138	61
Female	122	36	33	30	89	39
Median age (years)	63	28–87	66	41–87	62	28–83
Primum						
Colon	221	66	73	67	148	65
Rectal	111	33	32	30	79	35
T1	9	3	2	2	7	3
T2	45	13	18	17	27	12
T3	240	71	73	67	167	74
T4	28	8	11	10	17	8
Lymph node positive primary	202	60	58	53	144	63
Median CEA (ng/ml)	11.1	0–4858	6.8	0.7–3800	12.9	0–4858
Median number of metastases	2.0	1–12	1.0	1–10	3.0	1–12
Mean diameter of the largest metastasis (cm)	3.9	0.1–23	3.5	0.1–12	4.6	0.1–23
Synchronous	182	54	44	40	138	61
Metachronous	154	46	65	60	89	39
Median DFI (months, 154 patients)	15	0–180	16	1–180	15	0–81
<12 months	229	68	64	59	165	73
<24 months	284	86	86	79	198	87
Resectable extrahepatic metastases	39	12	15	14	24	11
Median OS (months)	51	95% CI 42–60	54	95% CI 38–70	50	95% CI 38–62
Median DFS (months)	10	95% CI 8–12	14	95% CI 7–21	9	95% CI 7–11

*NAC* neoadjuvant chemotherapy, *CEA* carcinembryonic antigen, *DFI* disease free interval, time between resection of primary cancer and diagnosis of liver metastases, *OS* overall survival, *DFS* disease-free survival, *CI* confidence interval

Patients with NAC had a significantly higher median number of metastases (3 vs. 1, *p* = < 0.0001), higher median CEA levels (12.9 vs. 6.8 ng/ml, *p* = 0.023), and suffered more often from synchronous liver metastatic disease (60.7 vs. 40.4 %, *p* = 0.0007). A statistically not significant higher number of pN1 primary tumors (65.6 vs. 56 %; *p* = 0.093) and larger diameters of metastases (4.6 vs. 3.5; *p* = 0.116) could be observed in patients with NAC.

The majority of patients with NAC (81.1 %, *n* = 184) had initially resectable CLM, whereas 18.9 % (*n* = 43) had initially unresectable disease.

Those with initially resectable disease received in 48.4 % (*n* = 89) NAC based on Oxaliplatin, in 10.9 % (*n* = 20) based on Irinotecan, in 36.4 % (*n* = 67) a combination with Avastin, and in 4.3 % (*n* = 8) combination with Cetuximab. Patients with initially unresectable disease received in 30.2 % (*n* = 13) NAC based on Oxaliplatin, in 7.0 % (*n* = 3) based on Irinotecan, in 41.9 % (*n* = 18) a combination with Avastin, and in 20.9 % (*n* = 9) a combination with Cetuximab.

Tumor-specific variables altered in patients with NAC compared with baseline values. There was a significant decrease in the median diameter of metastases (3.5 ± 3.3 cm vs. 2.0 ± 2.9 cm, *p* < 0.001) and in CEA levels (12.7 ± 458.0 vs. 6.6 ± 508.9 ng/ml; *p* < 0.001) after NAC, whereas the median number of metastases remained virtually unchanged (3.0 ± 2.1 vs. 2.0 ± 2.7; *p* = 0.462). During NAC, the diameter of the largest metastases decreased in 71.7 % (*n* = 162), remained the same in 8.4 % (*n* = 19), and increased in 19.9 % (*n* = 45). The CEA level decreased in 58.3 % (*n* = 127), remained the same in 8.7 % (*n* = 19), and increased in 33.0 % (*n* = 72).

Radiological imaging before initiation of NAC showed resectable extrahepatic metastases in 24 patients (10.6 %), whereas intraoperatively resectable extrahepatic metastases were found in 27 patients (11.9 %).

### Predictive Value of Clinical Scores in Patients without NAC

In patients without NAC, the Fong, Nordlinger, Nagashima, and Konopke scores could be calculated for 106, 103, 103, and 109 patients, respectively. Correlations of the CRSs with DFS and OS for patients without NAC are shown in Table 3. Regarding the prediction of DFS, Fong, Nordlinger, and Nagashima scores were able to show significant differences between the risk groups. However, only the Nagashima score was of predictive value for OS in patients without NAC.

**Table 3:** DFS and OS in patients without NAC and before/after NAC

	Without NAC				Before NAC				After NAC			
	Low	Intermediate	High	*p* value	Low	Intermediate	High	*p* value	Low	Intermediate	High	*p* value
DFS median	DFS	DFS	DFS		DFS	DFS	DFS		DFS	DFS	DFS	
Fong	21.0 (83)		8.0 (23)	**0.036**	14.0 (109)		6.0 (103)	**<0.001**	16.0 (120)		6.0 (92)	**<0.001**
Nordlinger	45.0 (27)	13.0 (63)	3.0 (13)	**0.002**	16.0 (40)	9.0 (135)	6.0 (43)	**0.001**	20.0 (45)	9.0 (142)	5.0 (31)	**<0.001**
Nagashima	47.0 (28)	10.0 (66)	13.0 (9)	**0.005**	15.0 (33)	11.0 (143)	5.0 (42)	**<0.001**	18.0 (46)	9.0 (139)	6.0 (33)	**<0.001**
Konopke	21.0 (53)	31.0 (41)	7.0 (15)	0.145	16.0 (65)	9.0 (87)	6.0 (65)	**<0.001**	17.0 (69)	8.0 (91)	5.0 (57)	**<0.001**

	Without NAC				Before NAC				After NAC			
	Low	Intermediate	High		Low	Intermediate	High		Low	Intermediate	High	
OS median	OS	OS	OS	*p* value	OS	OS	OS	*p* value	OS	OS	OS	*p* value
Fong	57.0 (83)		37.0 (23)	0.116	67.0 (109)		43.0 (103)	**0.026**	76.0 (120)		34.0 (92)	**<0.001**
Nordlinger	72.0 (27)	53.0 (63)	30.0 (13)	0.056	85.0 (40)	50.0 (135)	32.0 (43)	**<0.001**	86.0 (45)	49.0 (142)	28.0 (31)	**<0.001**
Nagashima	126.0 (28)	39.0 (66)	31.0 (9)	**0.001**	84.0 (33)	51.0 (143)	29.0 (42)	**<0.001**	84.0 (46)	51.0 (139)	20.0 (33)	**<0.001**
Konopke	53.0 (53)	58.0 (41)	35.0 (15)	0.454	67.0 (65)	49.0 (87)	44.0 (65)	0.173	71.0 (69)	59.0 (91)	31.0 (57)	**0.017**

Bold values are statistically significant

*DFS* disease-free survival, *OS* overall survival, () n

### Predictive Value of Clinical Scores in Patients with NAC

The Fong, Nordlinger, Nagashima, and Konopke scores could be calculated for 212, 218, 218, and 217 patients, respectively. Correlations of the CRSs with DFS and OS in patients with NAC are shown in Table 3.

Before and after NAC, all risk scores showed significant differences in DFS between the risk groups. Before NAC, Fong, Nordlinger, and Nagashima scores were of predictive value for OS. After NAC, all four CRSs showed significant differences in OS between the risk groups.

### Clinical Score Change During NAC

Overall, score increases during NAC were associated with a worsening in clinical outcome. The scores decreased, and therefore improved, in 27.4 % (*n* = 58, Fong) versus 23.9 % (*n* = 52, Nordlinger) versus 26.1 % (*n* = 57, Nagashima) versus 15.7 % (*n* = 34, Konopke). They remained the same in 63.2 % (*n* = 134, Fong) versus 67.0 % (*n* = 146, Nordlinger) versus 62.8 % (*n* = 137, Nagashima) versus 76.0 % (*n* = 165, Konopke). They increased in 9.4 % (*n* = 20, Fong) versus 9.2 % (*n* = 20, Nordlinger) versus 11.0 % (*n* = 24, Nagashima) versus 8.3 % (*n* = 18, Konopke).

**Fig. 1:**
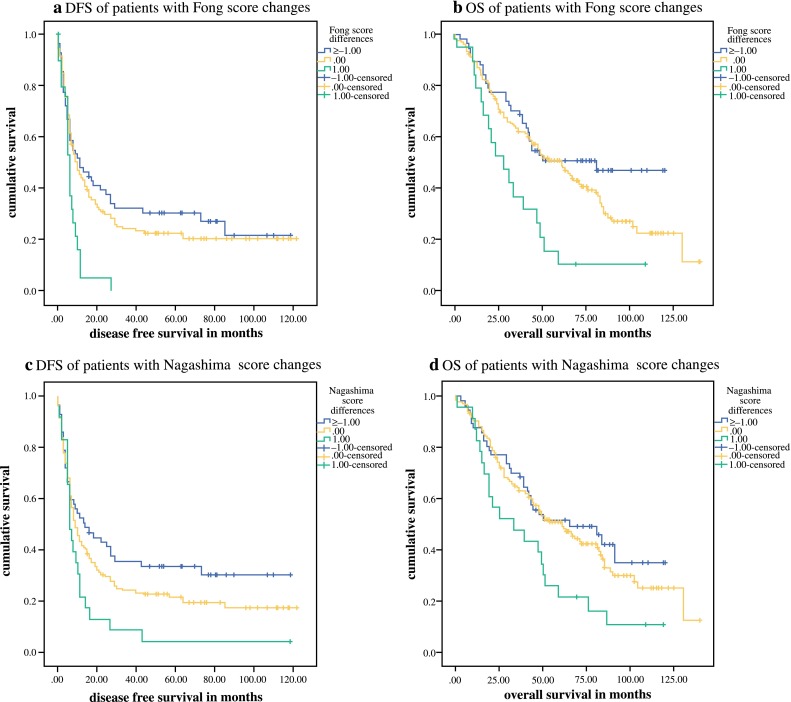
Kaplan–Meier curves of patients with increasing, steady or decreasing Fong and Nagashima scores

Whereas there was only a trend towards reduced survival in patients with increasing Nordlinger and Konopke scores, decreasing, steady, or increasing Fong and Nagashima scores had significant impact on DFS and OS (Table 4). Kaplan–Meier curves for patients with score changes of the Fong and Nagashima scores during NAC are shown in Figs. 1a–d.

**Table 4:** DFS and OS in patients with decreasing, steady or increasing scores during NAC

	≥−1	0	+1	
DFS median	DFS	DFS	DFS	*p* value
Δ Fong (*n* = 212)	11.0 (58)	10.0 (134)	6.0 (20)	**0.014**
Δ Nordlinger (*n* = 218)	9.0 (52)	10.0 (146)	5.0 (20)	0.131
Δ Nagashima (*n* = 218)	14.0 (57)	9.0 (137)	6.0 (24)	**0.012**
Δ Konopke (*n* = 217)	6.0 (34)	10.0 (165)	4.0 (18)	0.502

OS median	OS	OS	OS	*p* value
Δ Fong (*n* = 212)	81.0 (58)	61.0 (134)	28.0 (20)	**0.004**
Δ Nordlinger (*n* = 218)	72.0 (52)	51.0 (146)	30.0 (20)	0.109
Δ Nagashima (*n* = 218)	65.0 (57)	61.0 (137)	33.0 (24)	**0.032**
Δ Konopke (*n* = 217)	61.0 (34)	49.0 (165)	28.0 (18)	0.194

Bold values are statistically significant

*DFS* disease-free survival, *OS* overall survival, () n

## Discussion

Several clinical risk scores are used currently in clinical practice to predict prognosis after resection of CLM. Many of these were developed more than a decade ago when NAC was rarely applied in patients with CLM. Today, the consensus guidelines of the European Society for Medical Oncology recommend NAC as current standard in the management of potentially resectable liver metastases^10^. In patients with good prognosis and a single, small (<2 cm) liver metastases upfront surgery may be considered^10^.

The clinical practice guidelines of the National Comprehensive Cancer Network stated that in case of resectable synchronous liver and/or lung metastases only, upfront surgery as well as NAC followed by synchronous or staged colectomy and resection of the metastatic disease is recommended^11^. However, even though the number of patients receiving NAC is increasing, data on the predictive value of CRS in these patients are scarce.

We retrospectively assessed the predictive values of the Fong, Nordlinger, Nagashima, and Konopke scores in patients with and without NAC. The selection of the scores was based on the widespread clinical use of these scoring systems, the easily accessible data required for the calculation of these four scores, and the possibility to compare our findings with a previous study, published by Ayez et al. That study assessed the predictive value of these scores before and after NAC and concluded that the scores are not reliable when used to predict survival before the start of neoadjuvant treatment but are useful when calculated thereafter.

In patients with NAC, they found that only the Nagashima score regarding DFS and only the Nordlinger score regarding OS predicted outcome before and after NAC reliably^9^. A similar study reported that the Fong and the Nordlinger score could not predict OS in the setting of NAC^12^.

Regarding the reliability of risk scores in patients without NAC, controversial results were published in the recent literature^9^,^13^. Some studies demonstrated the usefulness of the Fong and Nordlinger scores to determine significant differences in DFS and OS^9^,^13^,^16^,^17^. However, another study contradicted these results^15^. Assessments of the Nagashima and Konopke scores regarding their accuracy to predict DFS and OS are rare, but some authors concluded that they seem to be useful tools^9^,^13^.

In the study population of Ayez et al., 193 patients received upfront surgery and 159 underwent NAC^9^. In comparison, we could include more patients with NAC but fewer patients without NAC. This might explain why we could observe more significant differences between risk groups in patients with NAC but also might have missed a significant difference in patients without NAC due to a lack of power.

Ayez et al. described a DFS of 9 months [95 % confidence interval (CI) 7–11] and an OS of 47 months (95 % CI 33–61) in patients with NAC. In our study population, similar median DFS (9 months, 95 % CI 7–11) and OS (50 months, 95 % CI 38–62) were observed.

In patients without NAC, Ayez et al. reported a median DFS of 14 months (95 % CI 11–17) and OS of 43 months (95 % CI 34–52). Our patients without NAC reached a similar median DFS (14 months, 95 % CI 7–21), whereas regarding OS, they performed markedly better (54 months, 95 % CI 38–70). As a reason, we assume that patients with good tumor biology and good prognosis more often underwent upfront surgery in our study population.

This selection bias in favor of upfront surgery for patients with favorable tumor biology and good prognostic clinical parameters might be a potential limitation of our study. We observed that patients who received NAC had a significantly higher median number and larger diameters of metastases, higher median CEA levels, and suffered more often from synchronous liver metastases and pN1 primary tumors. All of these parameters indicate a more aggressive metastatic disease. It is possible that scores of patients who received upfront surgery due to their good tumor biology are no longer predictive or at least of less predictive value.

A further limitation might be the inherent problem in retrospective analyses that must be taken into account when interpreting the results. However, the large sample size and the long follow-up might outweigh these limitations, particularly regarding the issues that the study was focused on.

Due to consistent accuracy of the Nagashima score through all our patient’s cohorts, it might be the most predictive. In contrast to the Konopke score, which was rather poorly performing across all our patients, the Nagashima score includes more variables. This probably allows for better patient characterization. Furthermore, patients are distinguished in three risk groups, which is probably more accurate than only two risk groups (Fong score). Indeed, the Nordlinger and the Nagashima score included similar variables and showed similar efficacy regarding overall outcome in our study. However, analyzing score changes during NAC, the Nagashima score was more predictive probably because this score included more changeable parameters than the Nordlinger score.

The assessment of the impact of score changes during NAC showed that only changes of Fong and Nagashima scores were associated with significant differences in survival. A reason for the poorer performance of the other scores might be that none of these CRSs exclusively used changeable parameters for calculation. For example, although the Nordlinger score considers six parameters, only two of them can be influenced by NAC. The Fong score changed in 36.8 %, the Nordlinger in 33.1 %, the Nagashima in 37.1 %, and the Konopke score in 24.0 %. Obviously, the Nordlinger and the Konopke are slightly more resistant to the influence of NAC. Additionally, even if parameters change during chemotherapy, this does not mean that they reach the cutoff values leading to score changes. Thus, to improve the impact of clinical scores during NAC on prediction of DFS and OS the use of continuous parameters should be considered.

In our institution, the use of CRSs did not influence the surgical management of CLMs. The process of selecting tailored therapy for each patient is still a goal of interdisciplinary conferences, including surgeons, oncologists, and radiologists. We believe that LR also should be offered to patients with high CRS; however, these patients might benefit from shorter follow-up intervals or an intensified NAC. Thus, further studies are needed to determine the outcome of high-risk patients and liver resection after NAC.

To conclude, only the Nagashima score was able to predict DFS and OS in all patient groups, irrespective of whether calculated before or after NAC. Calculation of changes of the Fong or the Nagashima score can be a valuable tool to estimate the clinical outcome of patients undergoing neoadjuvant treatment.
